# Newborn care practices among slum dwellers in Dhaka, Bangladesh: a quantitative and qualitative exploratory study

**DOI:** 10.1186/1471-2393-9-54

**Published:** 2009-11-17

**Authors:** Allisyn C Moran, Nuzhat Choudhury, Nazib Uz Zaman Khan, Zunaid Ahsan Karar, Tasnuva Wahed, Sabina Faiz Rashid, M Ashraful Alam

**Affiliations:** 1 Reproductive Health Unit, International Centre for Diarrhoeal Disease Research, Bangladesh (ICDDR,B), Dhaka, Bangladesh; 2 Department of International Health, Johns Hopkins Bloomberg School of Public Health, Baltimore, MD, USA; 3 Research and Evaluation Division, BRAC, Dhaka, Bangladesh; 4 Social and Behavioural Sciences Unit, International Centre for Diarrhoeal Disease Research, Bangladesh (ICDDR,B), Dhaka, Bangladesh; 5 World Bank, Dhaka, Bangladesh; 6 James P. Grant School of Public Health, BRAC University, Dhaka, Bangladesh

## Abstract

**Background:**

Urbanization is occurring at a rapid pace, especially in low-income countries. Dhaka, Bangladesh, is estimated to grow to 50 million by 2015, with 21 million living in urban slums. Although health services are available, neonatal mortality is higher in slum areas than in urban non-slum areas. The Manoshi program works to improve maternal, newborn, and child health in urban slums in Bangladesh. This paper describes newborn care practices in urban slums in Dhaka and provides program recommendations.

**Methods:**

A quantitative baseline survey was conducted in six urban slum areas to measure newborn care practices among recently delivered women (n = 1,256). Thirty-six in-depth semi-structured interviews were conducted to explore newborn care practices among currently pregnant women (n = 18) and women who had at least one delivery (n = 18).

**Results:**

In the baseline survey, the majority of women gave birth at home (84%). Most women reported having knowledge about drying the baby (64%), wrapping the baby after birth (59%), and cord care (46%). In the in-depth interviews, almost all women reported using sterilized instruments to cut the cord. Babies are typically bathed soon after birth to purify them from the birth process. There was extensive care given to the umbilical cord including massage and/or applying substances, as well as a variety of practices to keep the baby warm. Exclusive breastfeeding was rare; most women reported first giving their babies sweet water, honey and/or other foods.

**Conclusion:**

These reported newborn care practices are similar to those in rural areas of Bangladesh and to urban and rural areas in the South Asia region. There are several program implications. Educational messages to promote providing newborn care immediately after birth, using sterile thread, delaying bathing, and ensuring dry cord care and exclusive breastfeeding are needed. Programs in urban slum areas should also consider interventions to improve social support for women, especially first time mothers. These interventions may improve newborn survival and help achieve MDG4.

## Background

Urbanization is occurring at a rapid pace which has significant implications for maternal and child health. Globally, the urban population increased from 13% (220 million) in 1900 to 49% (3.2 billion) in 2005, and it is projected to grow to 60% (4.9 billion) by 2030 [1]. The majority of growth is in low-income countries; by 2050, it is estimated that 93% of global urbanization will occur in Asia and Africa [2]. This fast-paced growth is associated with the establishment of urban slums where crowded living conditions, poor sanitation, and widespread poverty are prevalent. Although health services are readily available in most urban areas, health indicators are generally worse in slum areas compared with urban non-slum areas. This rapid rate of urbanization coupled with the growth of urban slums will have a significant effect on the achievement of the Millennium Development Goals.

Bangladesh has experienced rapid urban growth. Dhaka, the capital city, had a population of 13 million in 2001 [3], and it is estimated to grow to 50 million by 2015 [4] of which almost half will live in urban slums [5]. Women in slum areas are more likely to give birth at home (88%) compared with women in urban non-slum areas (54%). In addition, there are differences in use of postpartum care; in urban non-slum areas, 42% of women reported a postnatal visit for their baby within two days of delivery, while in slum areas, only 13% of women reported this visit. There are also significant differences in newborn mortality. In Bangladesh as a whole, neonatal mortality is 37 per 1,000 live births, comprising 57% of under-five mortality [6]. Between 2002 and 2006, neonatal mortality was 43.7 per 1000 live births in slums compared with 20.1 per 1000 live births in non-slum areas [7]. It is therefore essential to address newborn health in urban slum areas to ensure achievement of the Millennium Development Goal 4 (MDG4) by 2015.

BRAC, a Bangladeshi NGO, initiated the Manoshi program in 2007, with funding from the Bill and Melinda Gates Foundation, to reduce maternal, neonatal and child death and morbidities among disadvantaged populations in urban slums of Bangladesh. This integrated comprehensive program provides a continuum of care from the community to referral facilities to address the major causes of maternal, newborn, and child disease and death.

Although fifty percent of neonatal mortality occurs in the first three days of life [8,9], there are few studies on health practices in urban slums, especially in regard to newborn care. The objective of this paper is to describe newborn care practices in urban slums in Dhaka, Bangladesh to inform Manoshi program interventions in an effort to improve newborn survival and achieve MDG4.

## Methods

This study utilized both qualitative and quantitative methods.

### Quantitative

A baseline survey was conducted in the Manoshi program and non-program slum areas in 2007. Respondents included women with a live birth in the year prior to the survey as well as women with children under five years of age. The objective was to measure selected maternal and newborn care practices as well as childhood immunizations to provide a baseline for the Manoshi program.

The Manoshi program included six slum areas (Gulshan, Shyampur, Kamrangir Char, Shabujbag, Mohammadpur, and Uttara). The non-program slum areas were chosen around the Manoshi program slums [4]. The sampling strategy followed a two-stage random cluster design. Each cluster comprised 175 households (an identifiable segment of a patty/block). In the first stage, fifty clusters from each area were selected by probability proportional to size in terms of slum population. To identify respondents, ten trained 'listers' visited each household in the selected clusters and listed the heads of the household, the address, and the availability of eligible respondents using a household listing form. In the second stage, women with a live birth in the last year and with a child under five years of age were randomly selected from the lists in Manoshi program and non-program areas.

The sample size for the baseline survey was based on improving maternal and newborn care practices including three or more antenatal care visits, facility delivery, postnatal care for the child, and child immunization. Baseline levels were estimated using the Bangladesh Urban Health Survey 2006 [7], and it was assumed that the program would achieve a 50% improvement in these indicators with a design effect of 1.5 (to adjust for the clustered sample). The required sample size ranged from 179 (3 or more antenatal care visits) to 628 (facility delivery). This estimate for facility delivery was based on a low level of facility delivery, and as Manoshi established delivery facilities for its catchment population, it was expected that the facility delivery indicator would be most affected during the program and that a smaller sample would be sufficient for assessing impact. Therefore, the sample included 600 women with a live birth in the last year and 600 women with a child under five. Trained interviewers collected information on reproductive history, knowledge and perception on pregnancy, delivery, post-partum and newborn care between August and October 2007. In this paper, results from the survey in the Manoshi study area are presented (n = 1,256) [10].

### Qualitative

To further explore newborn care practices, thirty-six in-depth semi-structured interviews were conducted in two slums in Dhaka city - Korail (n = 16) and Kamrangir Char (n = 20). The sample included both currently pregnant women (n = 18) and women who had at least one delivery (n = 18). Pregnant women were randomly selected from the Manoshi program's pregnancy register. Women with at least one delivery were purposively selected based on availability. Three interviewers with social science training interviewed the informants using a flexible interview guideline. The one-to-one interviews included questions on knowledge and practices around delivery and newborn care; they were tape-recorded. Fieldwork took place between March and October 2007.

Tape-recorded interviews were transcribed and coded in Bengali (Bangla), the local language, by the interviewers. A thematic approach was used to analyze the transcripts. The research team reviewed the transcripts to develop a code list for the topics related to the research questions. Codes were applied manually to the transcripts by the interviewers [11]. Text pertaining to the codes was organized in a matrix and translated into English. No software was used for the analysis.

This study was approved by the ICDDR,B Research Review and Ethical Review Committees. All women gave informed consent prior to completing the quantitative survey and qualitative interviews.

## Results

The background characteristics of respondents in the baseline survey are presented in Table 1.

**Table 1:** Background characteristics of the sample from the baseline survey

Characteristic	n	%
Electricity in household	1117	89
Piped drinking water in household	700	56
Ever attended school/madrassa	648	52
Religion		
Islam	1237	99
Hinduism	18	1
Buddhism	0	0
Christianity	1	0
Marital status		
Currently married	1232	98
Divorced, separated, widowed	24	2
Currently employed	313	25
Number of children ever born		
1-2	706	56
3-4	383	31
5 or more	167	13
**Total**	1256	100.0

Among women with a live birth in the year preceding the survey (n = 672), 84% gave birth at home assisted by a traditional birth attendant (TBA) (92%) or by a relative/neighbor (8%). Few women gave birth with a skilled provider (12%). Among all women, knowledge of immediate newborn care was mixed. More than half of respondents had knowledge about drying the baby (64%), wrapping the baby with warm clothes after birth (59%), and cord care (46%), while few women reported knowledge on eye care (1%).

### Cord care practices

There are many traditional practices around cutting the umbilical cord and caring for the umbilical cord stump. In the baseline survey, almost all women reported taking special care of the umbilical cord (98%). These practices were explored in the in-depth interviews.

In the in-depth interviews, the majority of women reported cutting the umbilical cord after expulsion of placenta, which ranged from 2 to 20 minutes after the birth of the baby. The timing of cutting the cord was important to the respondents; if the cord was cut before delivery of the placenta, it was perceived that the placenta could harm the woman's health by moving into her chest.

*We heard from our village's elder people that the cord shouldn't be cut before expulsion of the placenta. If they cut the cord before the placenta is expelled, it would be very painful. Sometimes the mother would die*. (Woman from Kamrangir Char)

Almost all respondents reported using a new or boiled instrument (blade or scissors) to cut the umbilical cord. Tying the umbilical cord with thread was found to be widely practiced. All respondents reported tying the cord twice with a gap between the abdomen and the first tie and between ties. Respondents reported that this method of tying the cord prevented bleeding. Few women reported boiling the thread used to cut the cord. The main reason for not boiling the thread was lack of awareness of the risks associated with using non-sterile thread.

*Tie the cord in two places with thread. The tie must be tight so that there will be no bleeding from the umbilicus stump. If there is bleeding, then it would create a problem. So the tie must be tight and then cut the cord after expulsion of the placenta*. (Woman from Korail)

In most cases, the traditional birth attendant (TBA) tied and cut the umbilical cord after delivery of the placenta. Other persons present in the delivery room - mainly elderly female family members and occasionally neighbours - assisted the TBA in preparing equipment for the birth. They helped the TBA to boil the blade and the thread, in cases where the thread was boiled. A few women reported cutting their baby's umbilical cord; this practice may reflect their home region.

*We are from Mymenshing district. In our village, the mother has to cut the cord after delivery. If the dai *[TBA] *or someone else cuts the cord, she would not able to pray for the next 41 days. Therefore, I had to cut the cord *[myself]. (Woman from Korail)

After cutting the cord, women reported cleaning the umbilical stump and its surrounding area, applying substances to the umbilical stump, and providing heat massage on and around the stump. These practices are performed with the ultimate goal of facilitating the drying and separation of the umbilical stump.

The women mentioned a range of substances applied to the umbilical stump including: mustard oil, mustard with chopped smashed garlic, coconut oil, boric powder, talcum powder, savlon (an antiseptic liquid) and *chular mati *(earth from a clay oven). These materials are usually applied until the umbilical stump dries up. Table 2 includes the substances reported as well as the reason for their use.

**Table 2:** Substances used on/around umbilical stump of the newborn babies in two Dhaka slums

Substance(s) used	Reasons for use
Boric powder or mustard oil with garlic on the stump	To dry up the umbilical stump and prevent infection
Warm mustard oil, coconut oil, or talcum powder, on the stump	To dry up the stump
Savlon on the stump	To prevent infection
*Chular mati *(earth from the clay woven) around the stump	To quickly dry up the umbilical stump To prevent discharge from the stump
Homeopathic medicine	To clean the stump To dry up the stump

Applying heat massage (*shek dewa*) to the umbilical stump and surrounding area was an integral part of home-based routine newborn care and was almost universally practiced among respondents. There are two prominent ways of applying heat massage. First, respondents reported holding a ball of soft cloth close to the fire and placing it on the umbilical stump until the cloth became cool. The caregiver checked the temperature of the cloth to ensure that it was tolerable for the baby prior to application. The second method includes putting one's thumb on or close to the chimney of a kerosene lantern and then placing the hot thumb on the newborn's umbilical stump.

The process is repeated for 10-15 minutes at a time one to three times per day until the cord falls off - between 3 to 40 days of life. The main reasons for applying heat massage are to facilitate the drying up of the stump and to reduce pain. Respondents also cited using heat massage to prevent discharge and umbilical stump infection as well as to give the umbilical stump a proper shape.

Some women who had contact with Manoshi staff reported avoiding heat massage because it was not recommended by the program. One woman in Kamrangir Char stated:

*We didn't apply sek deya *[anything]... *doctor advised that there is no need to give any sek deya. So I didn't give any sek. I saw many people given sek with coal but I am not doing that*. (Woman from Kamrangir Char)

Cleaning the umbilical stump is also an essential element of newborn care. The stump was usually cleaned with a piece of wet cloth and soap or antiseptic liquid. Dettol soap - an antiseptic soap available in the local market - was commonly mentioned. The following quote illustrates the range of activities used to clean the umbilical stump in the Dhaka slums.

*Before [the baby's] bath, I massage mustard oil all over the body of the baby. Then I apply some mustard oil around the umbilical stump. During [the] bath, with a wet cloth soaked with soap, I clean the umbilical stump. At night I apply "shek" *[heat massage] *on the umbilical stump with a *[heated] *folded cloth. Sometimes I wipe off the umbilical stump with dettol-mixed water*. (Woman from Kamrangir Char)

### Bathing and cleaning baby

In the baseline survey, 86% of women reported bathing the baby within the first two days after birth. This finding was supported by the in-depth interviews.

Most women reported bathing the baby soon after delivery, with most babies bathed just after the cutting of the umbilical cord. Delivery fluids and blood are regarded as polluted (*napak*) and hence the baby is not perceived to be clean or pure until it is bathed.

*After delivery and cutting the cord the baby was wiped off with a piece of cloth and kept on the bed. Then water was put in a bowl and the baby was bathed with soap. The baby was put in the bowl and slowly water was poured on its body*. (Woman from Korail)

*No one can take the baby on the lap because the baby has delivery blood on its body. If one touches the baby, one will get suit laga *[become polluted]. *Seniors can't take the baby on their lap because they can't pray. So the baby is bathed just after birth*. (Woman from Korail)

We found strong evidence of rigorous efforts to remove the vernix during the first bath and afterwards. The vernix was generally perceived as "filthy"; a product from the mother's womb that needed to be removed as soon as possible after birth.

*Those *[vernix] *are the filthy things the baby gets from the mother's womb. It looks ugly if that *[vernix] *is not removed, the skin looks dry*. (Woman from Korail)

*I rubbed off those *[vernix] *after birth. I rubbed properly with a piece of cloth. It was winter so I did not bathe my baby. I was afraid of cold so I wipe off the body. After three days I bathed the baby*. (Woman from Korail)

Women reported regular bathing of their babies during the first week of life, usually between two to seven times. Women reported trying to collect clean water to bathe their babies, and if the baby was sick, the number of baths was less frequent. The first bath was given with slightly warm water, and many women reported including dettol and/or soap. Mothers perceived dettol as having more antiseptic 'power' than normal bath soaps. Some women reported including other materials in the bath water such as raw turmeric and grass. One woman mentioned dipping a silver and gold ornament into the baby's bath water, as she perceived that this ornament would purify the water.

*I bathed the baby daily. There is a variation between persons in terms of the practice of bathing the baby. There are some women who get the baby cold during the bathing. They don't understand how to bathe *[a newborn baby] *properly. I bathe my baby in a way so that it does not get cold. The water is warm and I add savlon or mustard oil to the water*. (Woman from Korail)

The risk of cold was the main factor in determining the time of the bath. Women mentioned some measures to prevent their newborn babies from cold including mustard oil massage (before and after the bath) and wrapping the baby after the bath.

### Breastfeeding

In the baseline survey, 64% of women reported first feeding their baby something other than breastmilk (colostrum). Forty percent of women reported first giving their baby honey, while 16% of women gave sugar water, and 4% gave mustard oil. Half of women reported breastfeeding within one hour of birth, 35% reported breastfeeding within one day of birth, and 14% reported breastfeeding after one day of birth.

These findings were supported by the in-depth interviews. The majority of women first gave their babies either honey or sugar water, followed with breastmilk three to five days after birth. There are two dominant reasons for this practice. First, there is a preference for first giving the baby water or sweet food (honey). It is perceived that these foods will influence the child's personality and protect it from diseases like *mukhe gha *(oral thrush). Secondly, women perceived having insufficient milk until three to five days post-delivery. To ensure that their baby had sufficient food, they often supplemented with cow's milk, powdered milk, or sweet water. Some women mentioned putting mustard oil in the baby's mouth to clean *bizla bizla *(mucus) before feeding the baby honey.

*I learned this from seniors that the mouth of the baby remains dry after birth. If you give honey it works like moisturizer. The baby can take a gulp easily. ... If you feed the baby something sweet first then the baby will speak sweetly*. (Woman from Korail)

*For the first three days after birth there was no milk in my breasts. The baby sucked a lot but got no milk. The baby cried in hunger. Then I gave the baby tal misri water *[sugar water] *and had it suck my breasts. After three days there was sufficient breast milk and I did not feed anything except breast milk*. (Woman from Korail)

Women widely knew about colostrum and its benefits; however few women reported feeding colostrum to their babies. One woman explicitly mentioned that she threw away the colostrum and gave breast milk to her baby one and a half days after birth.

### Roles

The TBA was mainly responsible for taking care of the baby after birth. She held the baby, and in most cases, wiped and bathed the baby. The woman's mother and mother-in-law, if present, played an important role in attending the woman and the newborn baby. They held the baby after it was wiped and bathed.

The TBA's role was finished after the first day of birth. In urban slums, women often do not have many relatives nearby, and are therefore tasked with newborn care soon after birth. Women took care of the umbilical cord, first bath, cutting of the hair, and preparing food. Some women did report support from mothers, mothers-in-law, grandmothers, and neighbours. However, the majority of women reported conducting these activities on their own.

## Discussion

This paper presents reported newborn care practices among women in urban slums in Dhaka. The majority of women reported tying the cord in two places and using sterilized instruments to cut the cord after the delivery of the placenta. Babies are typically bathed soon after birth in an effort to purify them from the birth process. There is extensive care given to the umbilical cord including massage and/or substances applied to the umbilical stump, as well as a variety of practices to keep the baby warm. Exclusive breastfeeding is rare; most women reported first giving their babies sweet water, honey and/or other foods in addition to breastmilk. In general, these practices are similar to those in rural areas of Bangladesh and to urban and rural areas in other countries in the South Asia region [12-18].

These findings are based on self-reported newborn care practices, and may therefore differ from actual practices. However, given the consistency of findings in both the quantitative survey and qualitative interviews, we are confident that the findings represent actual practices. In addition, these studies were both conducted after the start of the Manoshi program. Therefore, some of the newborn care practices may have been influenced by the program.

In Bangladesh, Pakistan, and Nepal, the umbilical cord is cut soon after delivery, usually with an unsterilized blade or other sharp object. In the in-depth interviews in this study, almost all women reported using a sterilized blade, which is different than other studies conducted in rural areas of Bangladesh [15] and Nepal [16]. This practice may be due to increased access to shops and pharmacies and/or exposure to the Manoshi program. However, use of sterilized thread to tie the cord was less common, which is similar to other areas of Bangladesh [15] and rural Nepal [16]. Almost of respondents reported tying the umbilical cord in two places, mostly to prevent bleeding, which is uncommon in the South Asian literature [12,15,17]. This practice may be the result of living in an urban area and/or exposure to the Manoshi program.

The time immediately following delivery is a vulnerable period for both the woman and her baby. Appropriate management of the delivery of the placenta (or third stage of labour) is essential to prevent postpartum haemorrhage, the leading cause of maternal mortality globally and in Bangladesh. At the same time, the baby needs to be kept warm and dry to prevent hypothermia and infection, two of the major causes of neonatal mortality. In this study, women cited waiting until after delivery of the placenta before the umbilical cord was cut; ranging from 2 to 20 minutes, during which time the baby is often unattended. This is similar to other studies in rural Bangladesh, where the baby is left unattended until the delivery of the placenta [15,19-22].

Care of the umbilical cord is an important practice in most countries in South Asia. In Nepal, Pakistan, and Bangladesh, a variety of substances are placed on the cord to facilitate drying up and separation of the umbilical stump. In this study, women reported applying mustard oil, garlic, talcum powder, boric powder, savlon, and coconut oil on the umbilical cord stump. This practice has also been noted in rural areas of Bangladesh [15,19], Nepal [23] and Pakistan [18]. However, these practices can be harmful and may lead to infection. The World Health Organization recommends dry cord care (where no substances are placed on the umbilical cord) [24]. Other studies in Nepal have demonstrated reduced neonatal morbidity and mortality among infants whose umbilical stump was treated with chlorhexidine solution, especially among low birth weight newborns; however, additional evidence is needed to examine the effectiveness of this intervention [25].

In South Asia, it is believed that the mother and her baby are polluted or dirty (*napak*) after birth [21,26-30]. Pollution is a result of the birthing process, and it is perceived that the baby's risk of pollution can be removed by bathing [21]. This notion promotes immediate bathing of the baby and removal of the vernix, a protective covering. These behaviours often make the newborn more vulnerable to hypothermia and infection, which in turn influence the chance of neonatal mortality. The fear of cold is also prevalent in South Asia. Women and families go to great measures to keep the baby (and mother) warm in the days after delivery [21,31-33]. Mustard oil is often used to massage the baby to keep it warm in Bangladesh [14,21] and in Pakistan [34]. After the first bath, the baby is bathed less frequently, especially during the cold season, and baths typically include mustard oil or some other substance to help keep the baby warm. However, studies have demonstrated that repeated massage with mustard oil can be detrimental. In studies with mice, mustard oil decreases the skin's ability to act as a protective barrier [35]. Sunflower oil is a better alternative; it has been shown to improve skin barrier function [35] and reduce sepsis in pre-term babies in hospital-based studies [36,37].

It is also common in South Asia for the baby to be given sugar water or honey as its first food [12,15,17,18,23,34]. The notion that colostrum is "dirty" is widespread in the region, and often the colostrum is thrown away. It is perceived that sweet water or foods provide protection for the baby against illness and give the baby a sweet personality.

Although this study was conducted in an urban slum setting, the newborn care practices are similar to rural areas in South Asia, with the exception of tying the cord in two places and cutting the cord with a sterilized instrument. These findings therefore have program implications. First, the newborn is often left unattended for a significant period of time after birth. This is a vulnerable period, and there is a need to identify strategies to ensure adequate care via orientation for home-based birth attendants and/or identifying a newborn care person to be present at the birth. Second, messages to promote using sterile thread for tying the cord are essential, as well as additional messages on use of sterilized blades to cut the cord. Third, it is very important to keep the baby warm after delivery. Massage is a useful technique, but mustard oil may be harmful. Further research is needed on the feasibility, acceptability, and cost of using alternative oils, such as sunflower oil. Next, the umbilical cord should be kept dry and clean without applying other substances, and the baby should not be bathed until three days after delivery. Exclusive breastfeeding is essential; messages to deter the perception that colostrum is dirty and that women have insufficient milk supply need to be developed. The majority of these improved newborn practices can be successfully implemented at home. Comprehensive home-based newborn care programs in rural areas have been successfully implemented in India and Sylhet, Bangladesh with reductions of 62% and 33% of newborn mortality respectively [38-40]. Messages and interventions will need to be tailored to the urban context. This study also demonstrated that although newborn practices are similar in urban and rural areas, women have less social support in urban slums. Women received assistance from traditional birth attendants during delivery, but they were then left alone to care for their baby and themselves a few hours after delivery. Programs in urban areas, especially in urban slums, should consider interventions to provide social support to women, especially first time mothers, to ensure proper newborn care, breastfeeding, and maternal nutrition throughout the postpartum period.

## Conclusion

Urban slums are growing at a rapid rate in Bangladesh. Neonatal mortality is higher in slum compared with urban non-slum areas. There are a variety of potentially harmful newborn care practices in the urban slums of Bangladesh. This paper attempts to outline potential areas for program interventions to improve newborn survival in urban slums with the aim of achieving Millennium Development Goal 4.

## Competing interests

The authors declare that they have no competing interests.

## Authors' contributions

ACM, MAA, NC and SR conceived of the qualitative study, participated in its design, coordination, data collection, and data analysis. NUZK, NC, MAA conducted the data analysis and summarization of qualitative findings. TW assisted with the qualitative data analysis and finalization of the manuscript. ZAK participated in design of the quantitative study, coordination, data collection, and data analysis. ACM and MAA drafted and finalized the manuscript. All authors read and approved the final manuscript.

## Pre-publication history

The pre-publication history for this paper can be accessed here:

http://www.biomedcentral.com/1471-2393/9/54/prepub
